# Unveiling *Lodderomyces elongisporus* as an Emerging Yeast Pathogen: A Holistic Approach to Microbiological Diagnostic Strategies

**DOI:** 10.1007/s11046-024-00901-x

**Published:** 2024-10-28

**Authors:** Watcharamat Muangkaew, Natthapaninee Thanomsridetchai, Marut Tangwattanachuleeporn, Sumate Ampawong, Passanesh Sukphopetch

**Affiliations:** 1 Department of Microbiology and Immunology, Faculty of Tropical Medicine, Mahidol University, Bangkok, Thailand; 2 Faculty of Allied Health Sciences, Burapha University, Chon Buri, Thailand; 3 Department of Tropical Pathology, Faculty of Tropical Medicine, Mahidol University, Bangkok, Thailand

**Keywords:** *Lodderomyces elongisporus*, Elongated yeasts, *Saccharomyces elongisporus*

## Abstract

*Lodderomyces elongisporus*, first isolated in 1952, has increasingly been recognized as a significant pathogen, with a notable rise in human infections since the 1970s. Initially misidentified as *Candida parapsilosis* due to morphological similarities, *L. elongisporus* has now been conclusively established as a distinct species, largely due to advancements in molecular biology, particularly DNA sequencing. This review traces the detection history of *L. elongisporus*, from the earliest documented cases to the most recent reports, underscoring its role as a causative agent in human infections. It also explores therapeutic strategies that have demonstrated efficacy, alongside instances of environmental contamination reported in international literature. A critical evaluation of diagnostic methodologies essential for precise identification is provided, including culture-based techniques such as colony morphology on Sabouraud Dextrose Agar (SDA) and chromogenic media, coupled with microscopic assessments using Lactophenol Cotton Blue (LPCB) and Gram staining. The ultrastructure of *L. elongisporus*, as observed under Scanning Electron Microscopy (SEM), is also discussed. Furthermore, non-culture-based diagnostics, such as sugar utilization tests (API 20C AUX and the innovative in-house arabinose-based “Loddy” test) and antifungal susceptibility profiling, are reviewed, with a particular focus on molecular tools like ITS-DNA sequencing and MALDI-TOF MS, which, despite their higher costs, offer unparalleled specificity. The accurate distinction and characterization of *L. elongisporus* are paramount, particularly in vulnerable and immunocompromised patients, where misdiagnosis can lead to severe consequences. This review advocates for intensified research efforts to develop more accessible diagnostic tools and deepen our understanding of this emerging pathogen, ultimately aiming to improve patient outcomes.

## The significance and Intriguing Aspects of *Lodderomyces elongisporus*

The initial discovery and documentation of *Lodderomyces elongisporus* can be traced back to 1952, when a strain bearing similarities to *Candida parapsilosis* was isolated from citrus fruit [1, 2]. For an extended period, this organism was erroneously regarded as the teleomorph of *C. parapsilosis* due to their overlapping macroscopic characteristics. However, advancements in molecular diagnostics, particularly DNA sequencing of the 18S rRNA gene locus, have unequivocally established *L. elongisporus* as a distinct species. [3, 4]. Initially classified as *Saccharomyces elongisporus*, this organism was reclassified in 1966 to its current designation, *Lodderomyces elongisporus* [5]. By 1993, the species was identified in fruit juice, further cementing its recognition under the revised taxonomy [6].

**Fig. 1:**
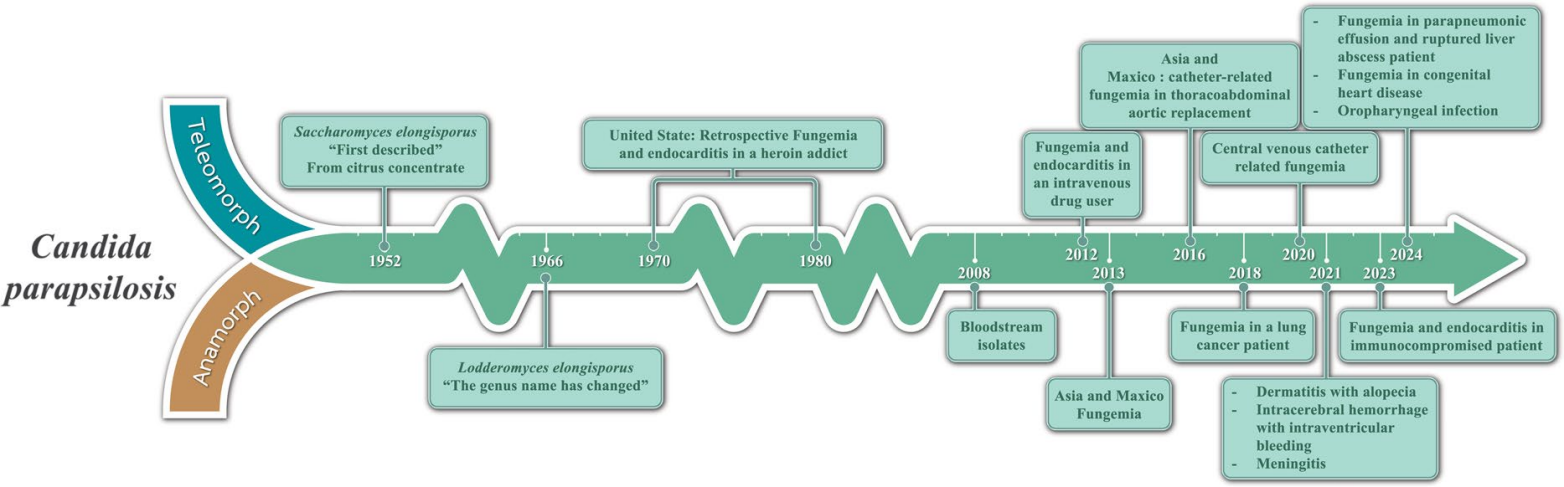
A historical overview of the discovery of *Lodderomyces elongisporus* and an analysis of case reports from past to present reveal that the majority of affected patients are immunocompromised, commonly presenting with symptoms of systemic mycosis (fungemia). Additional associated conditions include endocarditis, oropharyngeal infections, and meningitis

Since the 1970s and 1980s, the incidence of *L. elongisporus* infections in humans has seen a steady rise, a trend supported by retrospective analyses and a growing number of case reports. These infections have been documented across a broad demographic range, from neonates to elderly individuals over 50 years of age. The most severe cases predominantly affect vulnerable populations—especially the elderly, young children under 10 years old, and neonates—whose immune systems are compromised due to age-related factors or underlying medical conditions. Impairments in immune function, such as those associated with diabetes in elderly patients, low birth weight, or preterm delivery in newborns, significantly heighten susceptibility to fungal infections. In individuals aged 10–50 years, the risk is often exacerbated by factors including a history of intravenous drug use, recent surgical procedures, prolonged central venous catheterization, extended hospital stays, or immunosuppressive therapies or HIV/AIDS. These compounding factors underscore the urgent need for vigilant monitoring and advanced diagnostic strategies in at-risk populations, aiming to mitigate the heightened vulnerability and improve patient outcomes. Clinically, infections caused by *L. elongisporus* most commonly present as systemic mycoses or fungemia. However, there are also reports of less frequent but serious manifestations, including endocarditis, meningitis, oropharyngeal infections, and dermatitis (alopecia) (Fig. 1) [7–16].

A seminal review published in 2023 by Wang Y. and Xu J. highlights the global spread of *L. elongisporus*, with documented cases reported in at least 14 countries across five continents. Environmental sources of contamination have been identified in a diverse range of substrates, including citrus fruits, fruit juices, insects, fish, and hospital environments. Notably, *L. elongisporus* has also been isolated from pigeon droppings, which are recognized as a reservoir for the *Cryptococcus neoformans* species complex, particularly in samples collected from coastal regions. [1, 17].

This evolving understanding of this yeast highlights its emerging significance as a global pathogen with an expanding ecological presence and a growing impact on public health. The rising incidence of infections calls for heightened awareness, more focused research, and strategic interventions to mitigate the risks associated with this increasingly prevalent fungal species.

The treatment of infections caused by *Lodderomyces elongisporus* presents a fortunate scenario in clinical mycology, as this pathogen remains susceptible to all major classes of conventional antifungal agents. These include the Anti-Pyrimidine class (Flucytosine), Polyenes (Amphotericin B), Azoles (such as fluconazole, itraconazole, voriconazole, and posaconazole), and Echinocandins (including micafungin, caspofungin, and anidulafungin). Extensive research has demonstrated that these agents exhibit varying Minimal Inhibitory Concentrations (MICs) [1], highlighting the critical importance of tailoring the therapeutic regimen—specifically, the dosage, route of administration, and duration of therapy—to the individual needs of the patient. This personalized approach must be guided by the attending physician, who will consider the severity of the infection, the results of antifungal susceptibility testing, and the socioeconomic factors impacting both the healthcare facility and the patient.

Beyond the judicious selection of antifungal agents, prompt removal of indwelling devices becomes imperative when the infection is linked to surgical procedures or prolonged catheter use. Immediate removal of these devices, coupled with the application of topical antifungal and antibiotic treatments to affected areas, is essential to controlling the infection. Equally important is the thorough management of the patient’s underlying conditions. By addressing both the fungal infection and any comorbidities in an integrated manner, clinicians can significantly enhance the effectiveness of the treatment. The ultimate success in reducing the morbidity and mortality associated with *L. elongisporus* infections hinges on the rapid and accurate diagnosis of this pathogen. The forthcoming discussion will delve into the various diagnostic techniques available for identifying *L. elongisporus*, underscoring their pivotal role in guiding effective treatment strategies. The urgency and precision of these diagnostic approaches cannot be overstated, as they are the cornerstone of successful clinical outcomes in managing this emerging fungal threat.

Wang Y and Xu J (2023) highlighted the distinctive characteristics of *Lodderomyces elongisporus*, observing that this yeast predominantly exhibits elongated cell forms, in contrast to the typical budding yeast morphology commonly seen in *Candida* species. This unique cellular structure is notably reflected in the species name itself. Furthermore, at the genetic level, this fungus has a genome size of 15–16 Mb and is categorized within the CTG clade, where the CUG codon translates as serine instead of leucine. Despite its classification within this clade, *L. elongisporus* exhibits notably lower virulence compared to other members of the CTG clade, such as *Candida albicans* or *Candida parapsilosis*. This reduced virulence is characterized by its sensitivity to salinity, peroxide, and pH levels, its inability to transition to a mold or filamentous structure when phagocytosed by macrophages to evade innate immune cells, and its limited biofilm formation. Additionally, there is no clear evidence of sexual reproduction in *L. elongisporus*, as multiple ascospores in an ascus have not been observed, nor have the four mating type genes (MTLa1p, MTLa2p, MTLα1p, MTLα2p) been identified [1].

Beyond these fundamental biological characteristics, this review focuses on the microbiological laboratory findings associated with *L. elongisporus*. Our objective is to equip readers with an in-depth understanding that will advance accurate diagnostic approaches, ultimately leading to improved patient outcomes. The diagnostic techniques for this yeast are categorized into two main groups: culture-based and non-culture-based methods.

1. *Culture-based techniques*: These techniques predominantly focus on the observation of colony morphology, referred to as macroscopic findings, and are complemented by microscopic and ultrastructural examinations. These methods offer foundational insights into the phenotypic characteristics of the organism, providing initial clues for identification.
2. *Non-culture-based techniques*: This category includes an array of methods such as sugar utilization tests, drug susceptibility testing, and advanced molecular techniques. Among these, DNA sequencing and Matrix-Assisted Laser Desorption/Ionization-Time of Flight Mass Spectrometry (MALDI-TOF MS) stand out as powerful tools for precise species identification.

In addition to these established methods, this review introduces the in-house arabinose assimilation test, termed the Loddy test. Developed by our research team, the Loddy test is specifically designed to distinguish *L. elongisporus* from other *Candida* species with enhanced accuracy. This novel approach represents a significant advancement in the field, providing a more streamlined and effective diagnostic tool for mycologists.

## Culture-Based Diagnostic Techniques

Fungal species differentiation through culture-based methods primarily relies on the morphological characteristics of the fungi. This approach can be divided into two main types: macroscopic evaluation and microscopic evaluation.

### Macroscopic Evaluations

**Fig. 2:**
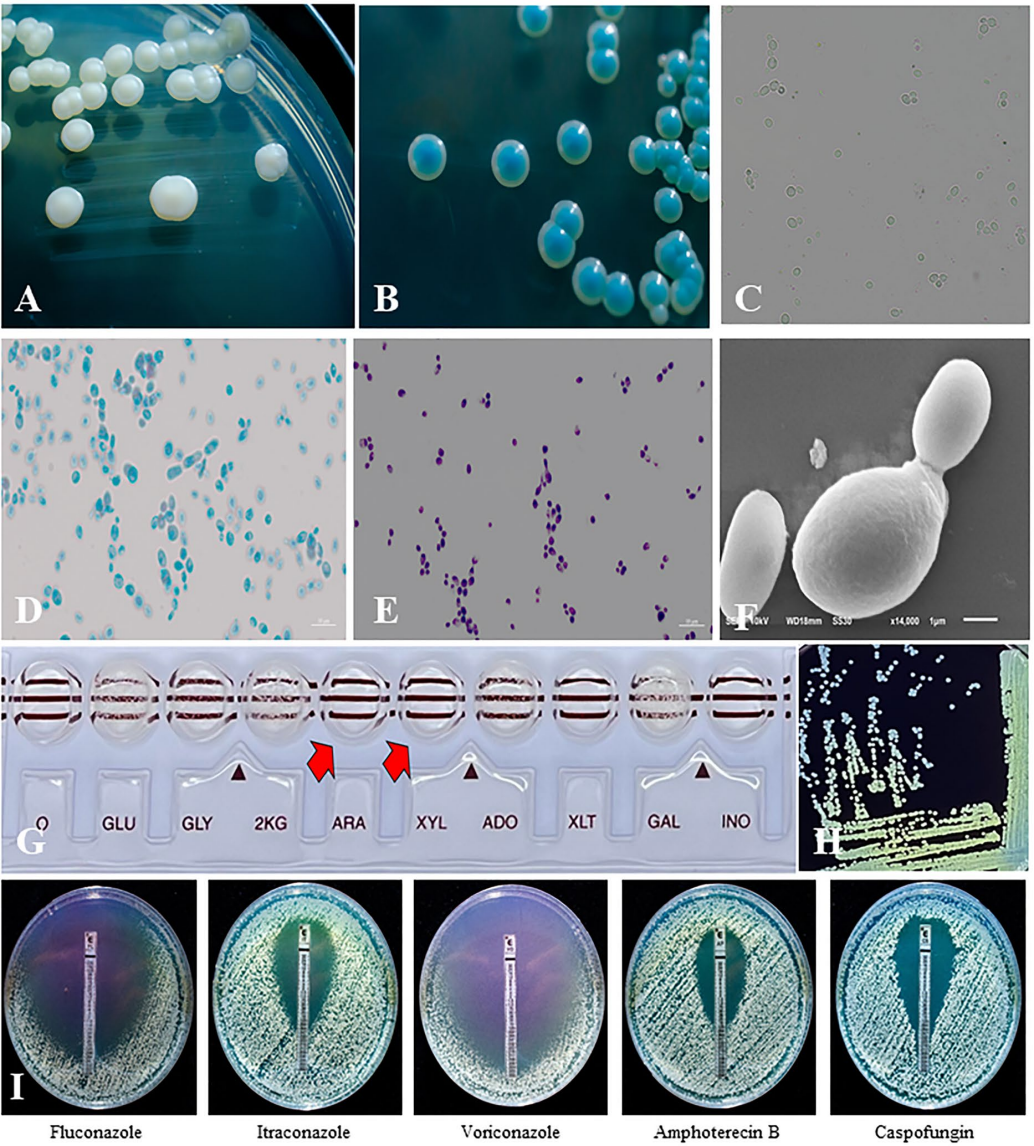
The identification of *Lodderomyces elongisporus* utilizes both culture and non-culture techniques. Culture-based methods include colony morphology examination on Sabouraud Dextrose Agar (**A** and Chromogenic Agar (**B**, along with microscopic analysis using Potassium Hydroxide (**C**, Lactophenol Cotton Blue (**D **Gram Stain (**E**, and Scanning Electron Microscopy (**F**. Non-culture methods involve sugar assimilation tests like API 20C AUX (**G**) and the Arabinose (Loddy) test (H), as well as antifungal susceptibility testing via the E-test (**I**)
- *Colony Color Testing on Chromogenic Agar for Yeast Isolation*: An effective approach for differentiating *Lodderomyces elongisporus* from other *Candida* species is the use of chromogenic agar, which leverages enzyme–substrate interactions. This specialized medium contains substrates that yield specific colors when they react with particular fungal enzymes, such as N-acetyl-hexosaminidase or alkaline phosphatase. For instance, *Candida albicans* typically produces a characteristic green color, while *Candida tropicalis* results in a metallic dark blue hue. In contrast, colonies of *L. elongisporus* display a distinctive blue-turquoise coloration, allowing for clear differentiation from other species. (Fig. 2B) [13, 18–20]. The precise enzymes responsible for the blue-turquoise coloration of *L. elongisporus* on chromogenic agar remain unidentified. This gap in knowledge presents a promising avenue for research, aimed at discovering the specific enzymes involved. Understanding these enzymes could greatly improve diagnostic accuracy and species differentiation in clinical settings, thereby refining diagnostic protocols and enhancing the precision of fungal identification in microbiological laboratories.

This method involves the phenotypic differentiation of yeast strains based on observable characteristics on culture media. Primarily, it focuses on evaluating colony morphology, including the shape, color, elevation, and edge characteristics of the colony, as well as the color change of the culture medium (reverse phase) and fungal growth kinetics. These macroscopic traits are critical for distinguishing between different yeast species. In microbiology laboratories, two commonly employed techniques include:

- *Colony Characterization on Sabouraud Dextrose Agar (SDA)*: In the cultivation of fungi on solid media, both general-purpose and selective media are utilized. For the isolation of yeast species, Sabouraud Dextrose Agar (SDA) and Potato Dextrose Agar (PDA) are among the most commonly employed media. These two media differ significantly in their nutrient compositions; SDA is characterized by its high dextrose content, while PDA is enriched with carbohydrates derived from potato infusion. Due to its high dextrose concentration, SDA is particularly favored for the isolation of yeast species. When cultured on SDA, *Lodderomyces elongisporus* displays a distinct colony morphology, characterized by a glossy, white-cream appearance. Notably, the colonies are more viscous and adhere strongly to the inoculation loop (Fig. 2A) [18, 19].

### Microscopic Evaluations

In this technique, the staining or clearing of obstructive cells is essential for accurate fungal diagnosis. A commonly used and straightforward bedside method involves the application of a 10% Potassium Hydroxide (KOH) solution. While KOH effectively stains fungal cells, it also renders the background transparent, which can sometimes obscure finer details of fungal structures. Being aware of this limitation is crucial for accurate interpretation of diagnostic results. The use of 10% KOH staining for diagnosing *Lodderomyces elongisporus* is not widely favored, as this method does not allow for precise yeast species differentiation. While it can reveal the presence of budding yeast, identifying the characteristic elongated yeast forms of *L. elongisporus* is often challenging with this stain. Additionally, patient samples infected with *L. elongisporus*, typically comprising blood or other bodily fluids, are more effectively cultured on solid media and subsequently examined using Lactophenol Cotton Blue (LPCB) staining or other more robust and reliable staining techniques. (Fig. 2C).

### Lactophenol Cotton Blue (LPCB) Staining

Various staining techniques are utilized in fungal diagnostics, each targeting the fungal cell wall. These include Lactophenol Cotton Blue (LPCB), Fuchsin Stains, Periodic Acid-Schiff (PAS) Stain, Congo Red, Aniline Blue, Uvitex 2B, and Calcofluor White, all of which produce distinct coloration. Among these, LPCB is particularly favored for its effectiveness and versatility. The principle behind LPCB staining lies in the affinity of cotton blue for the chitin and cellulose in fungal cell walls. This stain not only highlights the structural features crucial for fungal identification but also offers additional advantages: the lactic acid and phenol in LPCB help preserve and kill fungi, while glycerol prevents slide desiccation, creating semi-permanent mounts that allow for extended observation. After staining *Lodderomyces elongisporus* with LPCB, a key distinguishing characteristic is the significantly higher proportion of elongated budding yeast cells compared to other yeast species. Additionally, the conidia are slightly larger, typically measuring approximately 2–6 × 4–7 µm, which aids in its preliminary differentiation from other yeasts. (Fig. 2D) [17, 18, 20].

### Gram Stain (GS) for Fungal Staining

The Gram stain (GS) is primarily used to stain peptidoglycan, a major component of bacterial cell walls. However, when applied to fungal cells, the stain interacts with chitin, glucans, and polysaccharides found in fungal cell walls. Consequently, fungal cells typically appear blue-purple, similar to Gram-positive bacteria [18, 19]. Simultaneously, the presence of elongated yeast cells similar to those observed with LPCB staining can also be detected (Fig. 2E).

## Comparative Analysis of Yeast Surface and Morphology Using Scanning *Electron* Microscopy (SEM)

The application of Scanning Electron Microscopy (SEM) in fungal diagnostics is uncommon due to the complexity and labor-intensive nature of the procedure. SEM is predominantly employed to analyze fungal characteristics at the nanometer scale, excelling in determining particle size, shape, and texture, with the ability to examine objects as small as 1 nm or less. When SEM is utilized to differentiate *Lodderomyces elongisporus*, this yeast typically displays more elongated cells. A particularly notable feature observed through SEM is the uneven, wave-like surface texture, which contrasts with the smoother surface of other yeast species. The size is typically around 1.8–4.5 × 3.5–7.2 µm, consistent with findings from LPCB and Gram staining techniques (Fig. 2F) [4, 21].

The diagnosis and differentiation of *Lodderomyces elongisporus* from other yeasts using culture-based techniques—such as colony observation on Sabouraud Dextrose Agar (SDA), microscopic examinations with Gram stain and Lactophenol Cotton Blue (LPCB), and analysis under Scanning Electron Microscopy (SEM)—pose considerable challenges. Accurate identification often depends on the diagnostician’s expertise. However, chromogenic agar streamlines the process by producing distinct colony colors, with *L. elongisporus* displaying a characteristic blue-turquoise hue. Despite its specialized nature, this technique simplifies differentiation without necessitating advanced equipment or expertise.

### Non-Culture Based Diagnostic Techniques

Non-culture-based methods for fungal differentiation leverage the physiological traits of fungi, particularly variations in sugar utilization among yeast species. These methods enable precise testing and differentiation, facilitating accurate identification of various yeasts.

- *Sugar Utilization Testing with API 20C AUX*: The API 20C AUX system is a diagnostic kit used to evaluate the carbon source utilization properties of yeasts. This system consists of a strip with 19 wells, each containing a different carbon source. The carbon sources tested include Glucose, Glycerol, 2-Keto-D-Gluconate, L-Arabinose, D-Xylose, Adonitol, Xylitol, Galactose, Inositol, Sorbitol, α-Methyl-D-Glucoside, N-Acetyl-D-Glucosamine, Cellobiose, Lactose, Maltose, Sucrose, Trehalose, Melezitose, and Raffinose. When assessing the sugar utilization of *Lodderomyces elongisporus* with the API 20C AUX system, it is observed that *L. elongisporus* does not utilize L-Arabinose (Ara) or D-Xylose (Xyl) (Fig. 2G) [1, 11, 22]. This characteristic of not utilizing L-Arabinose led to the development of the in-house Arabinose (Loddy) test, specifically designed to differentiate *L. elongisporus* from other *Candida* species.
- *Arabinose (Ara) Utilization Testing with the In-house Arabinose (Loddy) Test*: Based on the characteristic non-utilization of Arabinose (Ara) by *Lodderomyces elongisporus*, the research team at the Faculty of Allied Health Sciences, Burapha University, developed a sugar utilization test kit known as the In-house Arabinose (Loddy) Test. This test has been validated with various *Candida* species, including *C. albicans*, *C. tropicalis*, and *C. parapsilosis*, all of which utilize Arabinose, yielding distinct results. The test kit is currently under consideration for patenting as the Loddy Test Kit for yeast arabinose utilization. The Loddy Test Kit is specifically designed to identify *L. elongisporus*. It demonstrates that this yeast, which does not utilize arabinose, does not cause a color change in the culture medium (Fig. 2H). In contrast, when testing *Candida* species that metabolize arabinose, the medium shifts to a golden yellow (data not shown).

Antifungal susceptibility testing, though not primarily for species identification, is vital for discerning drug resistance patterns, aiding clinical decision-making, and monitoring treatment. Its role in revealing resistance variations among species makes it indispensable in managing fungal infections.

### Antifungal Susceptibility Testing for Azole Drugs

Global reports increasingly underscore the challenge of antifungal resistance, particularly against standard azole drugs like fluconazole. However, *Lodderomyces elongisporus* remains susceptible to conventional antifungal agents, highlighting the critical importance of antifungal susceptibility testing. The E-test, which employs a plastic strip impregnated with an antifungal agent to determine the Minimal Inhibitory Concentration (MIC), enables rapid and accurate evaluation of drug efficacy, making it indispensable in laboratory mycology. Notably, *L. elongisporus* has demonstrated a favorable response to azoles, ensuring effective treatment protocols without the complications of drug-resistant strains (Fig. 2I) [23–26].

Both culture-based and non-culture-based techniques, along with antifungal susceptibility testing, demand substantial expertise in fungal diagnostics. Despite their utility, these methods can sometimes fall short in accurately identifying or differentiating fungal species. Consequently, advanced molecular techniques, such as Internal Transcribed Spacer (ITS) DNA sequencing and Matrix-Assisted Laser Desorption/Ionization-Time of Flight Mass Spectrometry (MALDI-TOF MS), have been increasingly adopted for their exceptional specificity. However, the high operational costs and specialized equipment required pose challenges for many healthcare facilities, necessitating a careful balance between precision and resource availability.

- *Internal Transcribed Spacer (ITS)-DNA Sequencing*: DNA sequencing is extensively employed to target various gene regions, including the Internal Transcribed Spacer (ITS), 18S rRNA (SSU rRNA), 28S rRNA (LSU rRNA), 5.8S rRNA, Beta-Tubulin (BT2), Elongation Factor 1-alpha (EF1-α), Calmodulin (CaM), Chitin Synthase (CHS), RNA Polymerase II Subunits (RPB1 and RPB2), and Actin (ACT) genes. Each gene offers distinct advantages and limitations. For yeast identification, ITS-DNA sequencing, particularly of the ITS1 and ITS2 regions, is the most prevalent method [17, 27–30]. These regions, which are part of the conserved ribosomal RNA genes, are highly effective in distinguishing various fungal species, particularly within the yeast group.

The sample, sample_2_Unknown_LE_contig-1, exhibited characteristics strongly indicative of Lodderomyces elongisporus. ITS-DNA sequencing confirmed its identity, providing definitive placement within the L. elongisporus group. This precise identification not only strengthened diagnostic confidence but also informed targeted treatment strategies. Despite its high cost and equipment requirements, ITS-DNA sequencing's unparalleled accuracy is invaluable, significantly enhancing both diagnostic precision and therapeutic outcomes.

## Matrix-Assisted Laser Desorption/Ionization-Time of Flight Mass Spectrometry (MALDI-TOF MS)

This tool analyzes the composition of sample substances by ionizing them and separating the resulting ions based on their mass-to-charge ratio (m/z), thereby generating a mass spectrum. This technique has been widely adopted for the analysis of pathogenic fungi from clinical specimens [17, 31-34]. The advantages of MALDI-TOF MS include rapid sample analysis and relatively lower operational costs compared to other methodologies. However, the initial investment in MALDI-TOF MS equipment, along with ongoing maintenance expenses, is substantial, limiting its accessibility in many healthcare facilities.

**Fig. 3:**
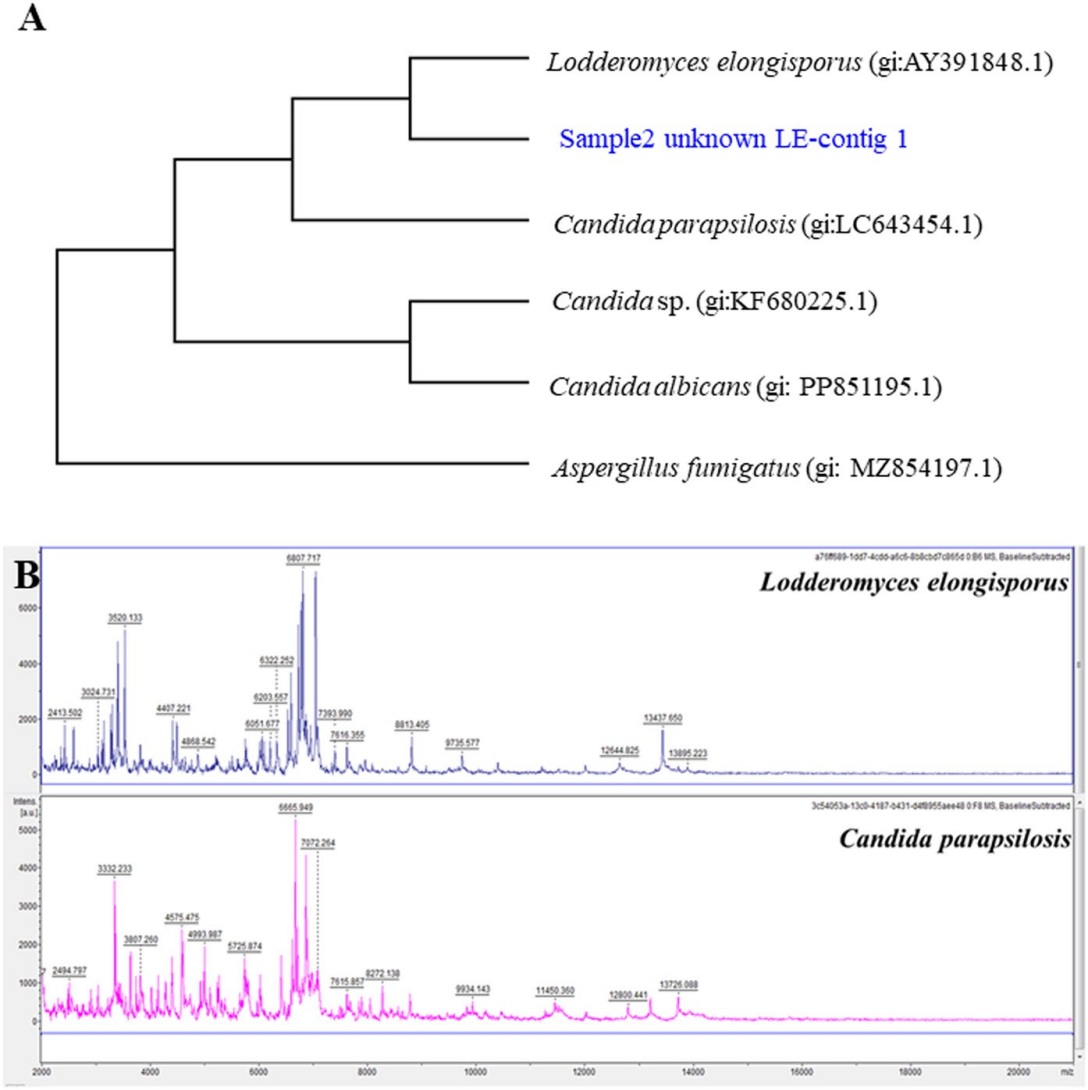
The application of molecular techniques for identifying and differentiating *Lodderomyces elongisporus* from other human pathogenic yeasts is crucial in clinical settings. The most commonly used methods are ITS-DNA sequencing (**A**) and Matrix-Assisted Laser Desorption/Ionization-Time of Flight Mass Spectrometry (MALDI-TOF MS) (**B**)

Moreover, MALDI-TOF MS excels in differentiating *Lodderomyces elongisporus* from *Candida parapsilosis*, species once mistakenly regarded as identical (Fig. 3B). This technique further distinguishes various yeast species by generating distinct mass spectra against established databases. However, its accuracy is limited when dealing with fungi that lack representation in these databases, often resulting in inconclusive or ‘‘unidentified fungus’’ outcomes.

### Transitioning From Laboratory Development to Advanced Research Initiatives

*Lodderomyces elongisporus* represents a formidable pathogenic challenge, yet critical knowledge gaps and diagnostic shortcomings, especially in resource-limited medical settings, remain unresolved. Bridging these gaps is not just essential but urgent for advancing infection control and deepening our understanding of its clinical implications. Targeted research is paramount to safeguarding patient health and significantly enhancing healthcare outcomes.

## Fundings

This review partially supported by Health Systems Research Institute (Grant number: HSRI 67–027) to Passanesh Sukphopetch.
